# Medical knowledge and clinical productivity: independently correlated metrics during radiology residency

**DOI:** 10.1007/s00330-020-07646-3

**Published:** 2021-01-15

**Authors:** Zahraa S. A. Alkhalaf, Derya Yakar, Jan Cees de Groot, Rudi A. J. O. Dierckx, Thomas C. Kwee

**Affiliations:** Department of Radiology, Nuclear Medicine and Molecular Imaging, Medical Imaging Center, University Medical Center Groningen, University of Groningen, P.O. Box 30.001, 9700 RB Groningen, The Netherlands

**Keywords:** Educational measurement, Internship and residency, Knowledge, Radiology, Workload

## Abstract

**Objective:**

To determine the association between medical knowledge relevant to radiology practice (as measured by the Dutch radiology progress test [DRPT]) and clinical productivity during radiology residency.

**Methods:**

This study analyzed the results of 6 DRPTs and time period–matched clinical production points of radiology residents affiliated to a tertiary care academic medical center between 2013 and 2016. The Spearman correlation analysis was performed to determine the association between DRPT percentile scores and average daily clinical production points. Linear regression analyses were performed to determine the association of DRPT percentile scores with average daily clinical production points, adjusted for age and gender of the radiology resident, and postgraduate year.

**Results:**

Eighty-four DRPTs with time period–matched clinical production points were included. These 84 DRPTs were made by 29 radiology residents (18 males and 11 females) with a median age of 31 years (range: 26–38 years). The Spearman correlation coefficient between DRPT percentile scores and average daily clinical production points was 0.550 (95% confidence interval: 0.381–0.694) (*p* < 0.001), indicating a significant moderate positive association. On multivariate analysis, average daily clinical production points (β coefficient of 0.035, *p* = 0.003), female gender of the radiology resident (β coefficient of 12.690, *p* = 0.001), and postgraduate year (β coefficient of 10.179, *p* < 0.001) were significantly associated with DRPT percentile scores. These three independent variables achieved an adjusted *R*^2^ of 0.527.

**Conclusion:**

Clinical productivity is independently associated with medical knowledge relevant to radiology practice during radiology residency. These findings indicate that clinical productivity of a resident could be a potentially relevant metric in a radiology training program.

**Key Points:**

*• There is a significant moderate correlation between medical knowledge relevant to radiology practice and clinical productivity during radiology residency.*

*• Medical knowledge relevant to radiology practice remains independently associated with clinical productivity during radiology residency after adjustment for postgraduate year and gender.*

*• Clinical productivity of a resident may be regarded as a potentially relevant metric in a radiology training program.*

## Introduction

Medical knowledge relevant to radiology practice is one of the core competencies of a radiologist [1]. In The Netherlands, the progress of knowledge acquisition of radiology trainees during residency is assessed by means of a semi-annual examination [2]. This Dutch radiology progress test (DRPT) is compulsory for each radiology resident during all 5 postgraduate years of radiology training [2]. Because the DRPT is a progress test, all residents are simultaneously given the same test, regardless of their duration of training [2]. This form of longitudinal testing sets it apart from final or modular examinations that are offered to radiologists in training in other countries like the USA and the UK [3, 4].

An important component of a radiologist’s job is image reporting [5]. Radiologists are expected to report a certain volume of imaging examinations per time unit to meet clinical demand [6]. Of interest, the utilization of medical imaging keeps on increasing in the Western world [7, 8]. The total number of images for each examination has also increased over the years [9]. Meanwhile, the number of radiologists has not followed a similar upward trend in several countries including the USA and the UK [9, 10]. Therefore, clinical productivity requirements are expected to continue to rise for the foreseeable future.

Acquiring medical knowledge relevant to radiology practice and achieving clinical production skills can be considered important targets during radiology residency. The first can be measured with examinations such as the DRPT, and the latter can be measured with metrics such as relative value units or study ascribable times [11]. However, the association between knowledge acquisition and clinical productivity during radiology residency remains unclear. It is hypothesized that they are correlated. Clinical productivity may increase knowledge because a higher caseload increases exposure of a resident to instructive clinical problems, anatomy, and pathology. Readily available knowledge may also speed up clinical productivity. On the other hand, when workloads are too high, this may be at the expense of a resident’s quality performance and educational efficiency. In the current radiology training program in The Netherlands, the relevance of clinical productivity is less outspoken. On the contrary, concerns have been raised about high psychosocial workload of residents, which is also debated in other countries [12]. However, if medical knowledge relevant to radiology practice and clinical productivity appear to reinforce each other, both residents and radiologists who train residents may ascribe more value to the latter, and clinical productivity may evolve into a more relevant metric during residency.

The purpose of this study was therefore to determine the association between medical knowledge relevant to radiology practice (as measured by the DRPT) and clinical productivity during radiology residency.

## Materials and methods

### Study design

The local institutional review board approved this study and waived the requirement for informed consent (IRB number: 201800838). This retrospective study was performed in the University Medical Center Groningen, which is a tertiary care academic institution in the north-east of The Netherlands. All residents, who were in training at the department of radiology of the University Medical Center Groningen between April 2013 and April 2016, were eligible for inclusion. Residents without any available DRPT results, e.g., due to dispensation from participation, illness, or not being affiliated to the University Medical Center Groningen at the time of any DRPT, were excluded. Note that residents could apply at the examination committee of the DRPT for dispensation from participation for various reasons, such as attendance of a course or congress, holidays, leave, health issues, and other circumstances in personal life [2]. DRPT results and time period–matched clinical production points were also excluded if clinical production points were acquired on less than 20 corresponding working days, in order to avoid non-representative samples. All authors on this paper (D.Y., J.C. de G., R.A.J.O.D., and T.C.K.), with whom the residents may potentially have a dependent relationship, were blinded to the names of the residents in relationship to their DRPT results and clinical production points.

### DRPT

The semi-annual DRPT was held in the spring (April) and autumn (October or November) in all years from 2013 until 2016, except for the DRPT in the autumn of 2015 that was canceled due to technical computer-related problems at the central test location [2]. The 6 DRPTs that were analyzed for the purpose of this study (April 2013 to April 2016, except autumn 2015) each had 180–200 computer-based test items containing a mixture of imaged-based questions and textual questions without any images [2]. Some of the image-based questions also had volumetric datasets that could be scrolled through. The test items involved questions related to abdominal radiology, breast radiology, cardiac and thoracic radiology, interventional radiology, musculoskeletal radiology, neuroradiology and head-and-neck radiology, and pediatric radiology [2]. The DRPT in the spring of 2016 also contained questions on nuclear medicine and molecular radiology because of merging of the residency training programs of radiology and nuclear medicine in The Netherlands [2]. Answer formats included true-false, multiple choice, long-list menu, and drag and drop items, and marker placements on image datasets [2]. The DRPTs that were taken in 2013–2016 were used for formative assessment, in which the test results were merely used for feedback purposes [2]. Further details about the DRPT can be read elsewhere [2]. Percentile scores, which range from 1 to 99, and which indicate the percentage of scores in the entire group of radiology residents that are lower than an individual’s score, were extracted for all of the 6 DRPTs for each radiology resident. Note that, nationwide, around 350 radiology residents participated in each of the 6 DRPTs [2].

### Clinical production points

Clinical productivity was determined based on a nationally used scoring system that assigns points to each radiologic procedure taking into account complexity and time. Ten-point classes are used, varying from 3 to 120 points per radiologic procedure (3, 5, 10, 15, 20, 30, 45, 60, 90, and 120 points) [13]. For example, 3 points are assigned to a conventional radiographic examination of the wrist, 15 points to a fluoroscopic swallowing study, 20 points to a ultrasonographic examination of the abdomen, 45 points to a magnetic resonance examination of the liver, 90 points to a computed tomography (CT) scan of the chest and abdomen, and 120 points to a percutaneous transluminal angioplasty of a renal artery [13]. Note that junior residents were permitted to perform and report interventional procedures (under supervision) and that they were then credited with the whole allocation of clinical production points. For each radiology resident, the number of clinical production points was determined in the 6 months after each DRPT, using the radiological information system. Only clinical production points acquired during office hours on weekdays (8.00–17.00) were included. Note that there are no formal requirements for residents to attain a certain number of clinical production points during office hours on weekdays at any time of the year. The number of working days of each radiology resident in the 6 months after each DRPT was also determined, using archived schedules. Officially recorded time that was spent on non-clinical activities (such as teaching, education, and research), holidays, and sick leaves was excluded. Subsequently, the average daily clinical production points of each radiology resident were calculated in the 6 months after each DRPT. Clinical production points during evening, night, and weekend shifts were excluded because residents on call are expected to perform acute radiological procedures only, and the demand for acute radiological procedures during these times can be highly variable. Nevertheless, to provide an estimate of the additional clinical work by the residents that is not included in this study, the average of the clinical production points that were acquired by the residents during all weekend shifts between April 2016 and April 2020 was calculated. This average number was then divided by three (corresponding to the total number of residents who are on call during weekend shifts) to estimate the average clinical workload of a resident during a weekend shift.

### Statistical analysis

A Shapiro-Wilk test was used to assess for normal distribution of DRPT percentile scores and average daily clinical production points. The Pearson or Spearman correlation analysis (for normally and not normally distributed data, respectively) was then performed to determine the association between DRPT percentile scores and average daily clinical production points. Correlation coefficients of 0–0.19, 0.2–0.39, 0.40–0.59, 0.6–0.79, and 0.8–1 were considered to indicate very weak, weak, moderate, strong, and very strong associations, respectively. Linear regression analyses were performed to determine the association of DRPT percentile scores with average daily clinical production points, age and gender of the radiology resident, and postgraduate year. Variables with a *p* value less than 0.05 on univariate analysis were entered in a multivariate model, provided the variance influence factor (VIF) of each variable was less than 3. *p* values less than 0.05 were considered statistically significant. Statistical analyses were executed using the MedCalc version 17.2 Software (MedCalc).

## Results

### Residents

Fifty radiology residents were affiliated to our hospital at some time between April 2013 and April 2016. Of these 50 radiology residents, 16 were excluded because they were not yet in training in our hospital (although they had already been registered in our department’s administration) and one was excluded because of dispensation (for which no further reason was documented in our department’s records). None of these 17 excluded residents had any available DRPT results or clinical production points during the time frame of the study. The 33 remaining radiology residents made a total of 95 DRPTs. Eleven of these 95 DRPTs were excluded because clinical production points were acquired on less than 20 corresponding working days. Finally, 84 DRPTs with time period–matched clinical production points remained for inclusion. These 84 DRPTs were made by 29 radiology residents (18 males and 11 females) with a median age of 31 years (range: 26–38 years). The characteristics of these 29 radiology residents are displayed in Table 1.

**Table 1:** Characteristics of 29 radiology residents who made a total of 84 DRPTs

Variable	No.
Age (years)	31 (26–38)^a^
Gender (M/F)	18/11
PGY at the time of each DRPT	
• PGY-1	16
• PGY-2	21
• PGY-3	23
• PGY-4	18
• PGY-5	7
DRPT percentile scores (%)^b^	36 (1–91)^a^
Average daily clinical production points^c^	420 ± 177^d^

^a^Median with range between parentheses

^b^Considering all 6 DRPTs

^c^Considering all 6-month time periods after each of the 6 DRPTs

^d^Mean ± standard deviation

*PGY*, postgraduate year

### Association between DRPT results and clinical productivity

**Fig. 1:**
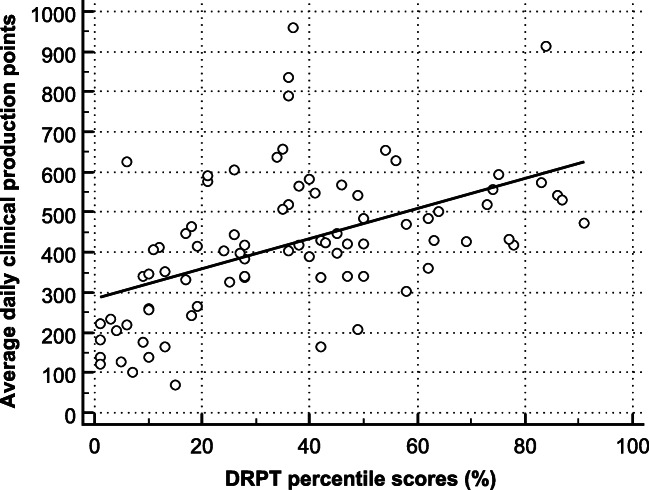
Scatterplot with DRPT percentile scores vs. average daily clinical production points, including regression line. The Spearman correlation coefficient was 0.550 (95% confidence interval: 0.381–0.694) (*p* < 0.001), indicating a moderate positive association

DRPT percentile scores were not normally distributed (*p* = 0.004), with a median of 36% (range: 1–91%). The average daily clinical production points were normally distributed (*p* = 0.060), with a mean ± SD of 420 ± 177 (range: 69.2–960.2). For comparison, the estimated average clinical workload or a resident during a weekend shift was 384 clinical production points (note that this number was not used in further analyses). The Spearman correlation coefficient between DRPT percentile scores and average daily clinical production points was 0.550 (95% confidence interval: 0.381–0.694) (*p* < 0.001), indicating a significant moderate positive association. A scatterplot with DRPT percentile scores vs. average daily clinical production points is shown in Fig. 1.

### Linear regression analyses

Average daily clinical production points, female gender of the radiology resident, and postgraduate year were significantly positively associated with DRPT percentile scores on univariate analysis (*p* < 0.001, *p* = 0.004, and *p* < 0.001, respectively), whereas age of the radiology resident was not (*p* = 0.448) (Table 2). On multivariate analysis, average daily clinical production points (β coefficient of 0.035, *p* = 0.003), female gender of the radiology resident (β coefficient of 12.690, *p* = 0.001), and postgraduate year (β coefficient of 10.179, *p* < 0.001) all remained significantly associated with higher DRPT percentile scores (Table 2). VIFs were 1.245, 1.008, and 1.242 for average daily clinical production points, female gender of the radiology resident, and postgraduate year, respectively, indicating no multicollinearity of the variables. These three independent variables achieved an adjusted *R*^2^ of 0.527.

**Table 2:** Univariate and multivariate linear regression analysis on the association of DRPT percentile scores with average daily clinical production points, age and gender of the radiology resident, and postgraduate year

Variable	Univariate analysis			Multivariate analysis		
	β coefficient	95% CI	*p* value	β coefficient	95% CI	*p* value
Average daily clinical production points	0.069	0.043 to 0.094	< 0.001	0.035	0.012 to 0.057	0.003
Age radiology resident	0.597	- 0.939 to 2.132	0.448	-	-	-
Female gender radiology resident	15.483	5.323 to 25.643	0.004	12.690	5.346 to 20.034	0.001
Postgraduate year	12.762	9.504 to 16.021	< 0.001	10.179	6.924 to 13.434	< 0.001

*CI*, confidence interval

## Discussion

The results of this study confirm our hypothesis that medical knowledge relevant to radiology practice is significantly correlated to clinical productivity during radiology residency. This correlation was moderate, which suggests that other factors also have independent effects on knowledge and clinical productivity. However, knowledge remained significantly and independently associated with clinical productivity on multivariate regression analysis. In the multivariate model, each unit increase in average daily clinical production points corresponded to a 0.035 increase in DRPT percentile score. In a simplified example, a resident with 285 more daily clinical production points than another resident on an average basis over time would theoretically have a 10-unit higher percentile score on the DRPT. For a resident doing a rotation in abdominal radiology, 285 clinical production points would, for example, correspond to 1 fluoroscopic swallowing study and 6 CT scans of the abdomen. It should be emphasized that this theoretical example is discussed for illustrative purposes only and may not be translatable to practice in which more variables affect test results. Moreover, it is more likely that there is a bidirectional causal relationship between radiological knowledge and clinical productivity, rather than that only one of them increases the other. Nevertheless, overall, these results indicate that clinical productivity should be considered important to measure progress during radiology residency. Based on these results, radiology training programs such as those in The Netherlands in which the main emphasis is currently on monitoring knowledge and practical skills acquisition may consider giving more significance to clinical productivity in performance reviews. Future studies are still required to establish clinical productivity benchmarks for this purpose, which should also take into account tolerable psychosocial workload limits of residents [12]. In addition, an important note should be made about the limitation of clinical production points as a stand-alone metric of productivity. In this study, the residents achieved an average daily clinical production points score of 420 (and an average of 384 clinical production points during weekend shifts). For a resident doing a rotation in abdominal radiology, 420 clinical production points would, for example, correspond to 1 fluoroscopic swallowing study and 9 CT scans of the abdomen. However, residents also spend time on many other important tasks than performing and interpreting radiologic procedures, which is not taken into account by the clinical production points. These other tasks include consultation with clinicians, protocolling imaging requests, preparing and participating in multidisciplinary meetings, supervision of younger residents and medical students, participation in educational and research activities, and some management tasks such as duty scheduling. Judging the performance of a resident solely on the basis of clinical production points should therefore be considered erroneous. Furthermore, it is plausible that exceeding a certain workload may be detrimental to a resident’s quality performance and educational efficiency. The fact that three out of four residents with the highest average daily clinical production points (i.e., 790 points and higher) had relatively low DRPT percentile scores (i.e., less than 40), as displayed in Fig. 1, feeds this hypothesis. The acceptable workload threshold is likely affected by characteristics of the resident (e.g., senior vs. junior trainees) and institutional factors (e.g., complexity of the workload), which requires further investigation.

A previous study by Ravesloot et al [14] investigated knowledge and image interpretation skill development during radiology residency by analyzing DRPTs performed between 2005 and 2010. No difference in expertise development was found between residents working in “academic” hospitals versus those working in “non-academic” hospitals [14]. This contradicted their hypothesis that residents in “non-academic” hospitals are exposed to a higher workload and image exposure, which would yield a more rapid image interpretation skill development. Ravesloot et al [14] speculated that a lower workload in “academic” hospitals allows for more in-depth study and receipt of feedback, and thus for deliberate practice and compensation for the lower image exposure in this setting. It was also speculated that the DRPT includes questions on less prevalent illnesses, which are rare in “non-academic” hospitals [14]. However, the study by Ravesloot et al [14] did not measure the effect of clinical productivity on expertise development directly, but indirectly inferred this by assuming that workload, in the sense of patient caseload, was higher in “non-academic” hospitals than in “academic” hospitals. The present study actually measured clinical productivity and showed that even in an “academic” setting, radiological knowledge is correlated with clinical productivity.

The results of the present study also showed knowledge relevant to radiology practice to be significantly and independently associated with a higher postgraduate year and female gender. The former is expected because it is the very essence of progress testing in which knowledge is expected to increase from novice to senior trainee [2, 14], and it supports the validity of our regression model. The latter is in line with the results of a previous study that reported women to generally outperform men on knowledge tests about clinical science concepts essential for patient care under supervision [15]. Of interest, another study has shown that females are better able to sustain their performance during a long test regardless of their relative advantage or disadvantage in the domain being assessed [16]. This was hypothesized to be due to the fact that males have been found to experience higher levels of boredom on activities with a long duration, which might cause impaired performance after some time of test-taking [16]. The DRPTs between 2013 and 2016 contained 180–200 computer-based test items that had to be answered within 2 h and 45 min [2]. As such, the DRPT can be considered a relatively long test that may potentially be of relative advantage to females. On the other hand, the study by Ravesloot et al [14] reported that gender did not influence expertise development during radiology residency. Furthermore, age was not found to be associated with DRPT results in the present study, but this also contradicts the findings in the study by Ravesloot et al [14]. Therefore, more research is necessary to determine the effect of gender and age on the development of knowledge during residency. More research is also necessary to identify other variables that affect knowledge acquisition of radiology trainees during residency, because only 52.7% of the variation in DRPT results could be explained by the factors that were investigated in the present study. Such information may potentially be used to further improve radiology training programs.

The present study had some limitations. First, due to its cross-sectional observational design, it was not possible to prove a causal relationship between medical knowledge relevant to radiology practice and clinical productivity or vice versa. This requires longitudinal research. Second, this study included data of residents from a single tertiary care academic institution. Future studies are required to confirm the generalizability of our results, including their applicability to “non-academic” settings. Third, the correlation between DRPT results and clinical productivity during evening, night, and weekend shifts was not analyzed, because the demand for acute radiological procedures during duty shifts can be highly variable. Nevertheless, the estimated average clinical workload of a resident during weekend shifts (384 clinical production points) was quite similar to the average daily production of a resident during office hours on weekdays (420 clinical production points). Fourth, although the clinical productivity of the residents was quantified, its quality could not be assessed. Fifth, it should be emphasized that this study did not investigate whether knowledge and clinical productivity during radiology residency are predictive of clinical productivity and quality of care during future professional practice as a radiologist. Nevertheless, in this context, it is of interest to note that a previous study by Walsh et al [17] reported a weak positive association between radiologist productivity and quality of resident teaching. The results of Walsh et al [16] and those of the present study suggest clinical productivity to be accompanied by other professional virtues in radiology practice.

In conclusion, clinical productivity is independently associated with medical knowledge relevant to radiology practice during radiology residency. These findings indicate that clinical productivity of a resident could be a potentially relevant metric in a radiology training program.

## Abbreviations

CT: Computed tomography
DRPT: Dutch radiology progress test
IRB: Institutional review board
PGY: Postgraduate year
VIF: Variance influence factor
